# Genetic Relationship Analysis of *Pastor roseus* Based on *COI* and *Cytb* Gene Sequences

**DOI:** 10.1002/ece3.72944

**Published:** 2026-01-14

**Authors:** Xixiu Sun, Xiaojie Wang, Ran Li, Huixia Liu, Ye Xu, Rong Ji, Jun Lin, Kun Yang, Xiaofang Ye

**Affiliations:** ^1^ International Research Center of Cross‐Border Pest Management in Central Asia, Xinjiang Key Laboratory of Special Species Conservation and Regulatory Biology, College of Life Sciences Xinjiang Normal University Urumqi Xinjiang Uygur Autonomous Region People's Republic of China; ^2^ Research Field (Migratory Biology), Observation and Research Station of Xinjiang Xinjiang Xinjiang Uygur Autonomous Region People's Republic of China; ^3^ Changji University Changji Xinjiang Uygur Autonomous Region People's Republic of China; ^4^ Center for Grassland Biological Disaster Prevention and Control of Xinjiang Uygur Autonomous Region Urumqi Xinjiang Uygur Autonomous Region People's Republic of China

**Keywords:** genetic diversity, genetic structure, mitochondrial DNA, *Pastor roseus*

## Abstract

This study aimed to elucidate the intra and interpopulation genetic variation of *Pastor roseus* in Xinjiang, China. Sequences of the mitochondrial genes *COI* and *Cytb* of 108 individuals from 10 distinct geographical populations across four regions of Xinjiang Uyghur Autonomous Region were analyzed. The mitochondrial genes were 1551 and 1143 bp in full length, respectively, and the AT content of bases was greater than the GC content. Based on the molecular variation in *COI* and *Cytb*, 62 and 69 haplotypes were detected, respectively; the average haplotype diversity (*H*_d_) values were 0.976 ± 0.006 and 0.944 ± 0.018, respectively, and the nucleotide diversity (π) values were 0.00316 ± 0.00016 and 0.00292 ± 0.00021, respectively, indicating that there was high genetic diversity among the 10 population. Analysis of molecular variance (AMOVA) indicated that the major source of genetic variation was within the populations. Analysis of molecular signatures of neutrality indicated that Tajima's *D* value was not significant, but Fu's *F*_S_ was significant, suggesting that *P. roseus* populations have recently experienced a large population expansion, but that the populations are currently relatively stable and the selection pressure is low, The Bayesian Skyline Plot (BSP) results indicate that the expansion occurred approximately 0.0015 million years ago. Although the *P. roseus* currently maintains a high level of genetic diversity and population connectivity, its recent population decline and the geographical isolation risks faced by some populations cannot be ignored. To sustain its crucial ecological role as a natural enemy of locusts, future conservation strategies should focus on ensuring habitat quality, preserving migration route integrity, and facilitating gene flow among populations.

## Graphical abstract

*Pastor roseus* exhibits exceptionally high mitochondrial diversity (haplotype diversity *H*_d_ > 0.94). The AT‐rich nucleotide composition in its *COI* and *Cytb* genes enhances energy metabolism efficiency during flight, while high haplotype diversity contributes to population resilience against environmental fluctuations. Although this study only examined mitochondrial genes *COI* and *Cytb*, future work should incorporate nuclear genomic markers (e.g., microsatellites) to provide a more comprehensive understanding of population genetic structure.

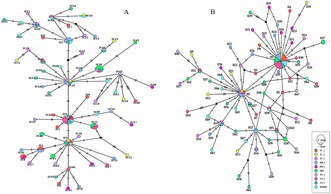

## 1 Introduction

*Pastor roseus* , a long‐distance migratory passerine of the family Sturnidae (Hobson and Yohannes 2007; Quader and Raza 2018), breeds annually with a population of 2–4 million individuals in locust‐prone grasslands of Xinjiang, at elevations ranging from 300 to 2500 m. Its reproductive cycle is highly synchronized with locust outbreak periods (Yu 2012). As an efficient natural predator of locusts, adults consume 120–180 locusts/day; it has achieved a long‐term locust control effect in a given area (Shi and Tan 2019), thereby protecting millions of hectares of grassland (Li et al. 2010). Genetic diversity provides a crucial adaptive foundation for avian migration, not only enabling birds to maintain high survival and reproductive success in the face of environmental changes, but also enhancing their predation efficiency (Lin 2024; Matthews et al. 2018). Populations that experience a loss of genetic variation may suffer reduced biocontrol capacity, while those with high genetic diversity exhibit enhanced predation efficiency, thus reducing pesticide residues from chemical interventions (Ranner et al. 1994; Frankham et al. 2002). In recent years, mitochondrial DNA (mtDNA) has emerged as a core molecular marker for investigating population genetic diversity and historical dynamics of aves due to its maternal inheritance, high mutation rate, and lack of recombination (Rahmadina and Tjong 2024). The high copy number of mtDNA enables efficient sequencing from trace samples such as feathers, feces, and blood, significantly promoting the technological progress of noninvasive sampling and large‐scale population genetic analysis (González‐Olvera et al. 2023). By analyzing haplotype diversity, constructing phylogenetic networks, and inferring population histories, mtDNA data can clearly reveal the phylogeographic patterns of species, the expansion and contraction events of populations, and assist in identifying conservation units with distinct evolutionary histories (Zink and Barrowclough 2008). In practice, mitochondrial genes such as *COI* and *Cytb* are frequently employed for species identification, phylogenetic reconstruction, and assessment of genetic differentiation due to their moderate evolutionary rates and high sequence conservation. These genes provide crucial genetic evidence for avian biodiversity research and conservation management (de Melo et al. 2021; Davidović et al. 2022).

The research on *P. roseus* in China has focused on technology to attract this species and understanding their locust control effect (Guo 2015), while research elsewhere has focused on changes in important physiological characters like water and energy metabolism during their migration (Milchev and Dimitrov 2005), as well as recording changes in its distribution (Kumar 2015; Diniarsih et al. 2016; Oo et al. 2020). To the best of our knowledge, no reports have been published on the population genetics of *P. roseus* . Accordingly, the present study analyzed the *COI* and *Cytb* gene sequences of *P. roseus* from various geographically separated populations in Xinjiang, determined their base compositions, and calculated various population genetic statistics. Ultimately, the phylogeographic relationships were elucidated among multiple *P. roseus* populations and individuals across different regions of Xinjiang, providing a preliminary assessment of the uniformity of *P. roseus* populations within this region. This study offers a scientific foundation for devising effective measures to conserve and utilize *P. roseus* germplasm resources.

## 2 Material and Methods

### 2.1 Sampling and Treatment

**FIGURE 1:**
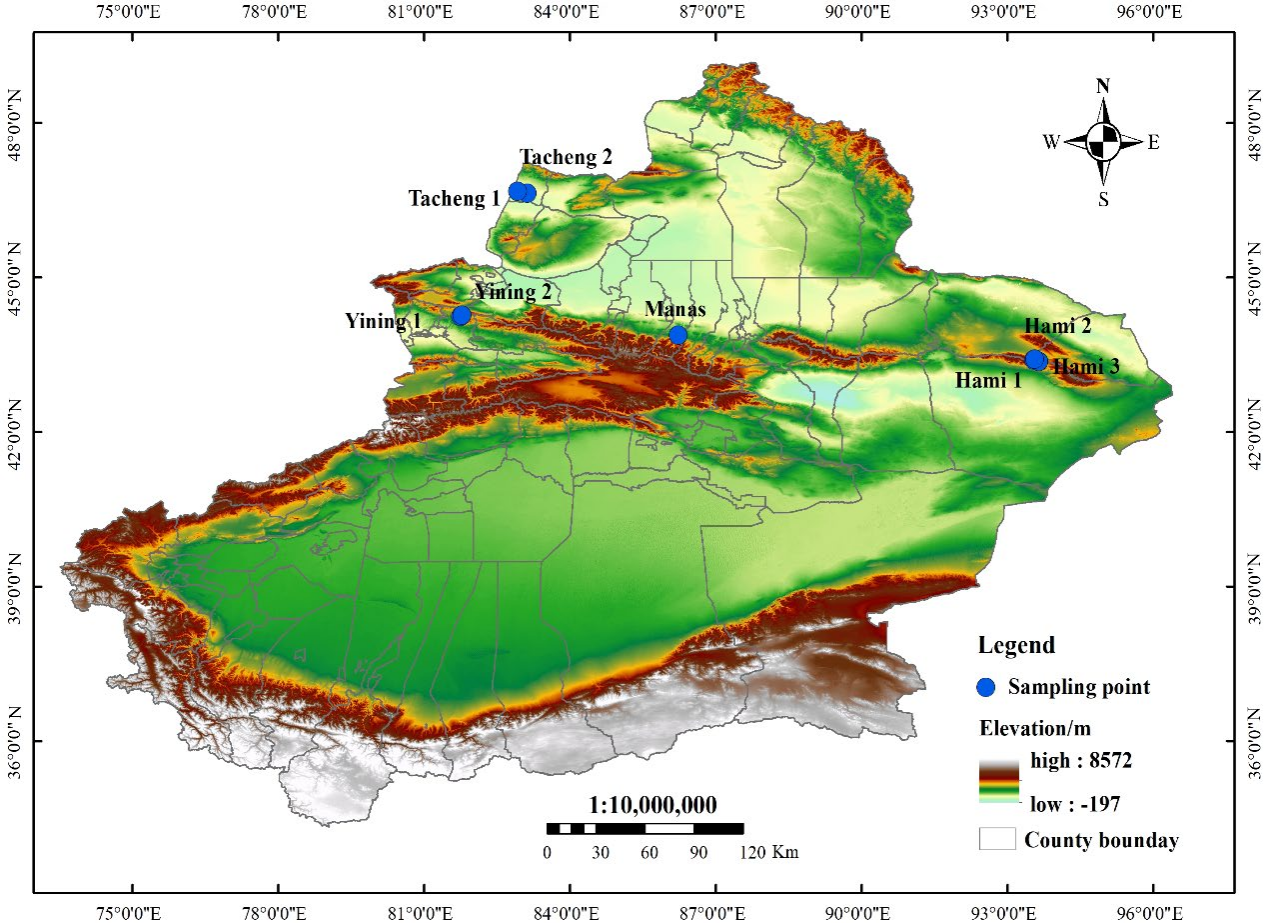
Distribution map of sampling sites.

Blood samples of *P. roseus* chicks from Hami City, Tacheng City, Manas County, and Yining County in Xinjiang, China were collected from May to July of both 2024 and 2025 (Figure 1 and Table 1). Multiple nests were randomly selected in the aforementioned areas, and the nests and chicks in them were labeled. Venous blood (0.5 mL) from the wing of one chick in each nest was collected and quickly transferred to a centrifuge tube with 1% heparin sodium solution. The mixture in the tube was thoroughly mixed by slowly pipetting it and then stored at −80°C.

**TABLE 1:** Sample information.

Group	Nest no.	Collection year	Corresponding to the collection site (Abbreviation)	Longitude and latitude
2025TC‐1	2025TC‐A1‐A12	2025	Tacheng City—Timber Mill (Tacheng 1)	46°38′38″ N, 83°6′33″ E
2025TC‐2	2025TC‐B1‐B7	2025	Nest 1, Innovation Road, Tacheng City (Tacheng 2)	46°40′40″ N, 82°54′43″ E
2025TC‐3	2025TC‐C1‐C10	2025	Nest 2, Innovation Road, Tacheng City (Tacheng 2)	46°40′40″ N, 82°54′43″ E
2025HM‐1	2025HM‐D1‐D15	2025	Hami City—Songshutang Community (Hami 2)	43°22′22″ N, 93°38′7″ E
2025HM‐2	2025HM‐E1‐E6	2025	Beside the Sheepfold, Third Company, Horse Ranch, Hami City (Hami 3)	43°25′25″ N, 93°33′3″ E
2025MNS	2025MNS‐F1‐F11	2025	Manas County—Provincial Highway 101	43°52′52″ N, 86°13′9″ E
2025YN‐1	2025YN‐G1‐G8	2025	Nest 1 at the Entrance of Tuohulasu Scenic Area, Yining County (Yining 1)	44°15′15″ N, 81°44′34″ E
2025YN‐2	2025YN‐H1‐H13	2025	Nest 2 at the Entrance of Tuohulasu Scenic Area, Yining County (Yining 1)	44°15′15″ N, 81°44′34″ E
2025YN‐3	2025YN‐I1‐I14	2025	Yining County—Artificial Stone Nests in Tuohulasu Scenic Area (Yining 2)	44°16′16″ N, 81°46′18″ E
2024HM	2024HM‐J1‐J12	2024	Hami City—Horse Ranch Company 3 Nurbai (Hami 1)	43°24′24″ N, 93°31′51″ E

*Note:* In the column of Nest No., A1‐A12, B1‐B7, C1‐C10, D1‐D15, E1‐E6, F1‐F11, G1‐G8, H1‐H13, I1‐I14, and J1‐J12 represent the number of *Pastor roseus* collected at each sampling point.

### 2.2 DNA Extraction, Amplification, and Sequencing

Total DNA was extracted from the blood samples using the Ezup Column Blood Genomic DNA Extraction Kit (B518253, Shanghai Biotechnology, Shanghai, China). Based on the mitochondrial *COI* and *Cytb* sequences of *P. roseus* , respective primers were designed using Primer Premier 5.0 software (Premier Biosoft, San Francisco, CA, USA) and synthesized by Shanghai Sangon Bioengineering Co. Ltd. (Shanghai, China) (Table 2).

**TABLE 2:** Primer information.

Primers	Primer sequences	Amplified fragment length
*COI ‐F* *COI ‐R*	AAAGGACTACAGCCTAACGC ACTAACACCTCTATGAGAAAGAAGC	1672
*Cytb‐F* *Cytb‐R*	ACCTCCACCACTCTCCACTC AAATGCCAGCTTTGGGAGTTG	1374

The PCR reaction volume (25 μL) contained 1 μL each of upstream and downstream primers, 9.5 μL of ddH_2_O, 12.5 μL of 2× Taq PCR Premix II, and 1 μL of total DNA template. The thermal cycling conditions for *COI* were as follows: 94°C for 5 min; 30 cycles of 94°C for 30 s, 60°C for 30 s, and 72°C for 1 min 40 s; 72°C for 10 min; and held at 4°C thereafter. The thermal cycling conditions for *Cytb* were as follows: 94°C for 5 min; 30 cycles of 94°C for 30 s, 68°C for 30 s, 72°C for 1 min 22 s; 72°C for 10 min; and held at 4°C thereafter. The PCR products were detected using agarose gel electrophoresis and sent to Shanghai Sangon Bioengineering Co. Ltd. (China) for Sanger sequencing.

### 2.3 Bioinformatic Analysis

Sequencing results were assembled using DNASTAR Inc., Madison, WI, USA, and assembled sequences were manually proofread for possible errors by referring to the sequencing chromatograms. Each sequence was compared to the publicly available complete mitochondrial *COI* and *Cytb* sequences of *P. roseus* (GenBank accession number PP792558). If any base mutations were present, they were manually corrected as necessary by referring to the peak patterns in the ABI files. After validation, the sequences were aligned using MEGA 11 (Tamura et al. 2021) to analyze their nucleotide composition, conserved sites (C), variable sites (V), parsimony‐informative sites (Pi), singleton mutation sites (S), and the frequency of base usage at each codon position. DnaSP 6 (Rozas et al. 2017) software was used to distinguish haplotypes and calculate classical population genetic parameters such as haplotype diversity (*H*_d_), nucleotide diversity (π), and the average number of nucleotide differences in the population (*K*). Arlequin v3.5.2.2 (Excoffier and Lischer 2010) software was used to perform molecular variance analysis (AMOVA), calculate the genetic differentiation index (*F*_ST_), and conduct neutrality tests for the 10 populations. Haplotype network relationship maps for 10 geographic populations of *P. roseus* were constructed using PopART 1.7 (Leigh Jessica et al. 2015) software. BSPs were analyzed using BEAST 2.7.7 (Barido‐Sottani et al. 2018) to infer the historical dynamics and approximate time range of effective sample size (ESS) in genetic lineages. The tree‐first test was applied to select the coalescent Bayesian skyline model, specified as HKY + G. The molecular clock model employed a Strict clock. Visualization of the historical dynamic map of effective population size was achieved using Tracer v1.7.2 software (Rambaut et al. 2018). Taking the mutation rate of the Cytb sequence of the mitochondrial DNA gene in birds at 2.1% per million years as a reference (Voelker and Light 2011) to estimate the divergence times among geographic populations of the *P. roseus* .

## 3 Results

### 3.1 DNA Extraction and *COI* and *Cytb* Amplification and Sequences

**FIGURE 2:**
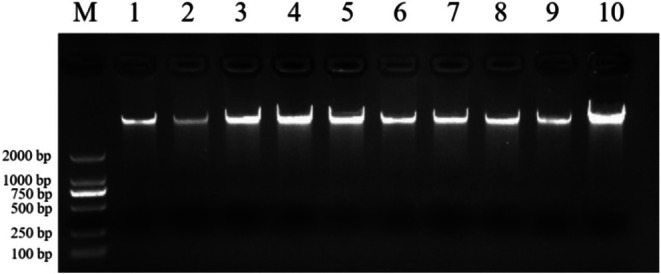
Gel diagram of total DNA extraction from *Pastor roseus* .

Genomic DNA was extracted from 108 blood samples of *P. roseus* from 10 geographic populations, and *COI* and *Cytb* mtDNA genes were amplified and sequenced. As shown in Figure 2, the extracted total DNA from each *P. roseus* sample had a single bright band without degradation. OD_260/280_ values of the sample were all between 1.8 and 2.0, indicating that the DNA was of good quality and could be used for subsequent experiments.

### 3.2 Base Composition

This study acquired 108 *COI* and *Cytb* sequences from *P. roseus* individuals, with sequence lengths of 1551 and 1143 bp, respectively. In the *COI* sequences, the average contents of the four bases A, T, C, and G were 27.96%, 23.38%, 31.49%, and 17.17%, respectively, with a slightly higher A + T content (51.34%) than C + G content (48.66%). Similarly, in the *Cytb* sequence, the average contents of A, T, C, and G were 28.80%, 24.11%, 33.98%, and 13.11%, respectively, with slightly higher A + T content (52.91%) than C + G content (47.09%; Table 3). Within the *COI* and *Cytb* sequences, G consistently exhibited the lowest content, indicating a pronounced anti‐G bias.

**TABLE 3:** Base composition of *COI* and *Cytb* gene sequences in *Pastor roseus* .

Codon site	*COI* base composition %				*Cytb* base composition %			
	A	T	C	G	A	T	C	G
The first	24.37	20.74	23.94	30.95	24.95	21.27	29.91	23.87
The second	18.19	39.85	26.88	15.09	20.48	40.67	26.26	12.59
The third	41.33	9.54	43.66	5.48	40.97	10.38	45.78	2.87
Average	27.96	23.38	31.49	17.17	28.80	24.11	33.98	13.11

The *COI* sequence contained 1477 conserved sites (C; representing 95.23% of the total sites) and 74 variable sites (V; constituting 4.77% of all sites). All *Cytb* sequences contained 1062 conserved sites (C; representing 92.91% of the total sites) and 81 variable sites (V; accounting for 7.09% of all sites). Among the *COI* and *Cytb* gene sequence codons, the first codon position exhibited the highest frequency of G and C, respectively (Table 3); the second codon position exhibited a considerably higher frequency of T than other bases, with G having the lowest frequency; the third codon position exhibited the highest frequency of C and the lowest frequency of G. The frequency of the four bases in the codons considerably differed, with G exhibiting the lowest frequency in the third codon position. In the *COI* and *Cytb* sequences of *P. roseus* , no insertions or deletions of abnormal fragments were observed, with most base substitutions being transitions and primarily occurring at the third codon position.

### 3.3 Population Genetic Diversity

DnaSP 6 was used to analyze the haplotypes, haplotype diversity, and nucleotide diversity of *COI* and *Cytb* gene sequences. From the *COI* gene sequences of *P. roseus* , 62 haplotypes were defined. The overall haplotype diversity, nucleotide diversity, and average nucleotide differences were 0.976 ± 0.006, 0.00316 ± 0.00016, and 4.896, respectively. From the *Cytb* gene sequences, 69 haplotypes were defined; the overall haplotype diversity, nucleotide diversity, and average nucleotide differences were 0.944 ± 0.018, 0.00292 ± 0.00021, and 3.334, respectively. Both gene sequences exhibited low nucleotide diversity and high haplotype diversity. The genetic diversity analysis of *P. roseus* in each population based on *COI* sequences (Table 4) revealed that the haplotype diversity of population 2025MNS was the lowest and those of populations 2025TC‐2, 2025TC‐3, 2025HM‐2, 2025YN‐1, and 2024HM were high. The genetic diversity analysis of *P. roseus* in each population based on *Cytb* sequences (Table 4) revealed that the haplotype diversity of population 2025YN‐2 was the lowest and those of populations 2025TC‐2, 2025TC‐3, 2025HM‐2, and 2025YN‐1 were high. The ratio of haplotype number to sample number of the population was 100%; that is, no individuals shared identical haplotypes.

**TABLE 4:** Genetic diversity index of 10 populations in *Pastor roseus* .

Gene	Populations	*n*	*N*	*H*_d_	*π*	*K*
*COI*	2025TC‐1	12	10	0.970 ± 0.044	0.00292 ± 0.00056	4.530
	2025TC‐2	7	7	1.000 ± 0.076	0.00461 ± 0.00073	7.143
	2025TC‐3	10	10	1.000 ± 0.045	0.00374 ± 0.00045	5.800
	2025HM‐1	15	13	0.981 ± 0.031	0.00321 ± 0.00031	4.971
	2025HM‐2	6	6	1.000 ± 0.096	0.00352 ± 0.00080	5.467
	2025MNS	11	7	0.909 ± 0.066	0.00241 ± 0.00032	3.745
	2025YN‐1	8	8	1.000 ± 0.063	0.00256 ± 0.00041	3.964
	2025YN‐2	13	9	0.910 ± 0.068	0.00231 ± 0.00058	3.590
	2025YN‐3	14	13	0.989 ± 0.031	0.00266 ± 0.00036	4.121
	2024HM	12	12	1.000 ± 0.034	0.00329 ± 0.00039	5.106
	Total	108	62	0.976 ± 0.006	0.00316 ± 0.00016	4.896
*Cytb*	2025TC‐1	12	8	0.848 ± 0.104	0.00199 ± 0.00060	2.273
	2025TC‐2	7	7	1.000 ± 0.076	0.00383 ± 0.00076	4.381
	2025TC‐3	10	10	1.000 ± 0.045	0.00387 ± 0.00059	4.422
	2025HM‐1	15	11	0.933 ± 0.054	0.00195 ± 0.00035	2.229
	2025HM‐2	6	6	1.000 ± 0.096	0.00292 ± 0.00047	3.333
	2025MNS	11	7	0.909 ± 0.066	0.00347 ± 0.00064	3.964
	2025YN‐1	8	8	1.000 ± 0.063	0.00366 ± 0.00063	4.179
	2025YN‐2	13	8	0.808 ± 0.113	0.00227 ± 0.00064	2.590
	2025YN‐3	14	13	0.989 ± 0.031	0.00319 ± 0.00041	3.648
	2024HM	12	10	0.955 ± 0.057	0.00241 ± 0.00034	2.758
	Total	108	69	0.944 ± 0.018	0.00292 ± 0.00021	3.334

*Note:* Here, *n* represents the number of *P. roseus* individuals samples at each sampling point; *N* represents the number of shared haplotypes; *π* represents nucleotide diversity. *H*_d_ represents haplotype diversity. *K* represents the average number of nucleotide differences.

### 3.4 Population Genetic Structure of *P. roseus*

Based on the gene sequences of *COI* and *Cytb* in *P. roseus* , the genetic differentiation index (*F*_ST_) and gene flow (*N*_m_) of 10 populations were calculated.

**FIGURE 3:**
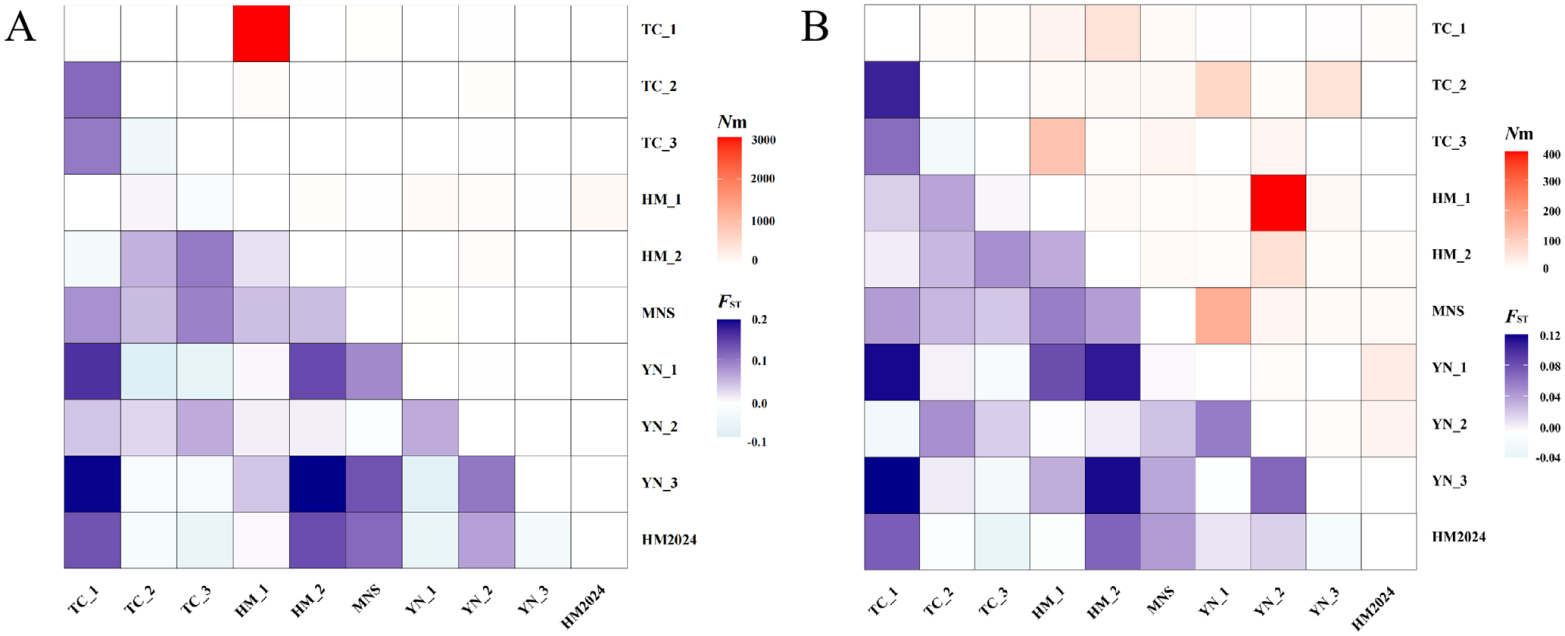
*F*_ST_ (lower diagonal) and *N*_m_ (upper diagonal) values between different geographical populations of *Pastor roseus* based on *COI* (A) and *Cytb* (B) gene sequences. TC_1 through YN_3 are all samples from 2025; HM2024 is a sample from 2024.

For *COI*, *F*_ST_ values among populations ranged from −0.07907 to 0.19085, with the smallest genetic differentiation between populations 2025YN‐1 and 2025TC‐2 and the greatest differentiation between populations 2025TC‐1 and 2025YN‐3. *N*_m_ values of the 10 populations ranged from 2.09741 to 2756.15, with the most frequent exchanges occurring between populations 2025HM‐1 and 2025TC‐1 (Figure 3A; Table S1).

For *Cytb*, *F*_ST_ values among populations ranged from −0.03198 to 0.12009, with the smallest genetic differentiation between populations 2025TC‐3 and HM2024 and the greatest genetic differentiation between populations 2025TC‐1 and 2025YN‐3. *N*_m_ values of the 10 populations ranged from 3.66348 to 396.2651, with the most frequent exchanges occurring between the 2025HM‐1 and 2025YN‐2 populations (Figure 3B; Table S1).

Molecular ANOVA of *COI* and *Cytb* gene sequences revealed that the intrapopulation variation was absolutely dominant. Among them, intrapopulation variation and interpopulation variation in *COI* sequences accounted for 95.4% and 4.6% of the total variation, respectively. Intrapopulation variation and interpopulation variation in *Cytb* sequences accounted for 96.61% and 3.39% of the total variation, respectively (Table 5).

**TABLE 5:** Analysis of molecular variation (AMOVA) of the two mitochondrial genes *COI* and *Cytb* between the 10 samples of *Pastor roseus* .

Marker	Source of variation	df	Sum of squares	Variance components	Percentage of variation
*COI*	Among populations	9	32.023	0.1131 Va	4.6
	Within populations	98	229.922	2.34614 Vb	95.4
	Total	107	261.944	2.45924	
*Cytb*	Among populations	9	20.004	0.05664 Va	3.39
	Within populations	98	158.339	1.61570 Vb	96.61
	Total	107	178.343	1.67234	

**FIGURE 4:**
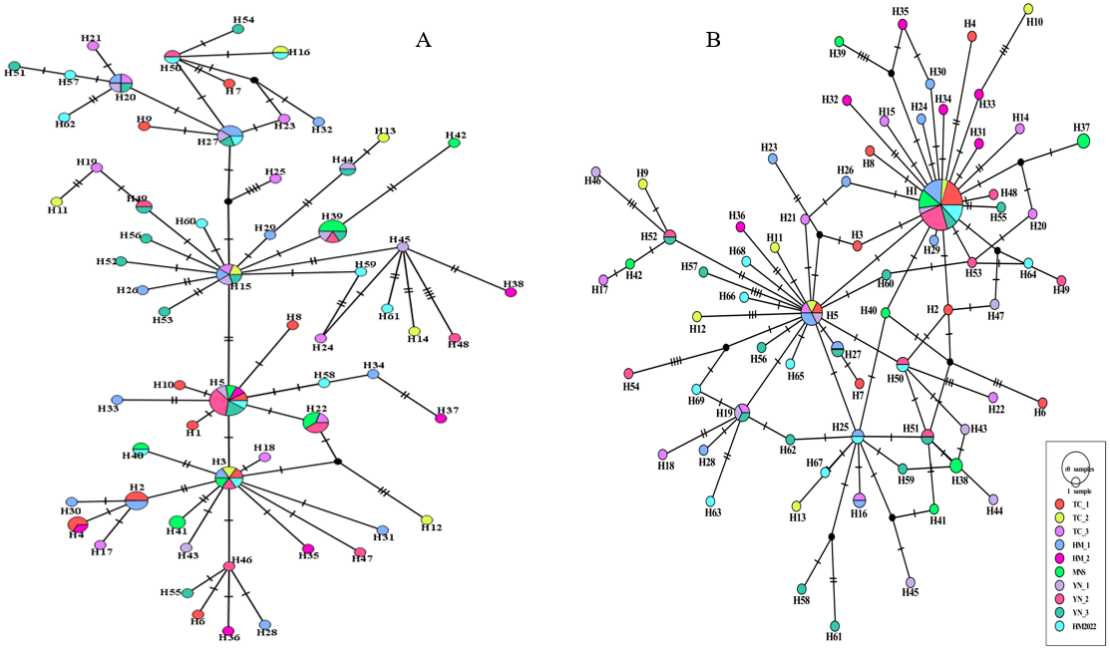
Haplotype network of *Pastor roseus* from 10 populations. (A) The network of *COI* sequences. (B) The network of *Cytb* sequences. Each circle represents a unique haplotype. Different colors represent the different 10 populations of *P. roseus* . The size of each circle is proportional to the number of haplotypes contained. The lines (shaded markers) on the branches indicate the mutations differentiating haplotypes, with each mutation represented as a line.

Haplotype network maps of *P. roseus* from 10 geographic populations based on *COI* and *Cytb* sequences were constructed. As shown in Figure 4, the haplotypes of both genes were relatively dispersed, and there were many haplotypes, indicating high genetic diversity among populations.

### 3.5 Historical Dynamics Analysis of *P. roseus* Population

**FIGURE 5:**
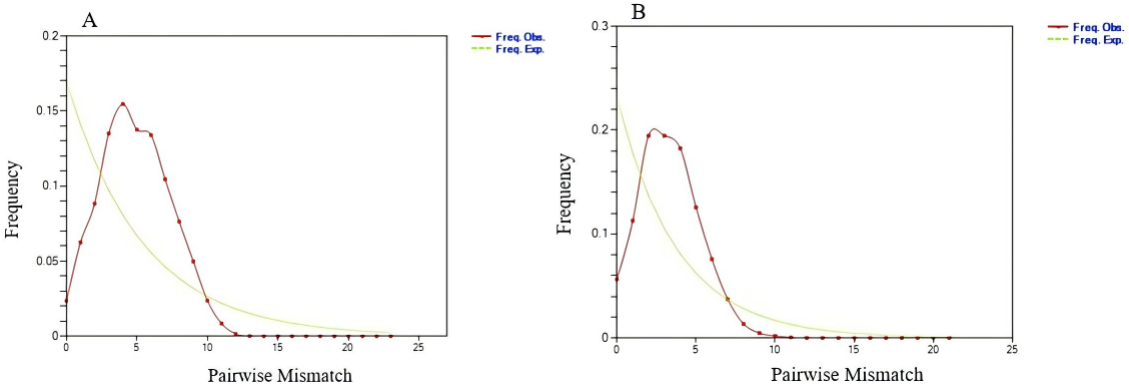
The mismatch analysis of 10 *Pastor roseus* populations based on *COI* and *Cytb* gene sequences. The *x*‐axis shows the number of pairwise differences, while the *y*‐axis shows the frequency of mismatches. Freq. Exp, frequency expected (green dashed line); Freq. Obs, frequency observed (red solid line).

**FIGURE 6:**
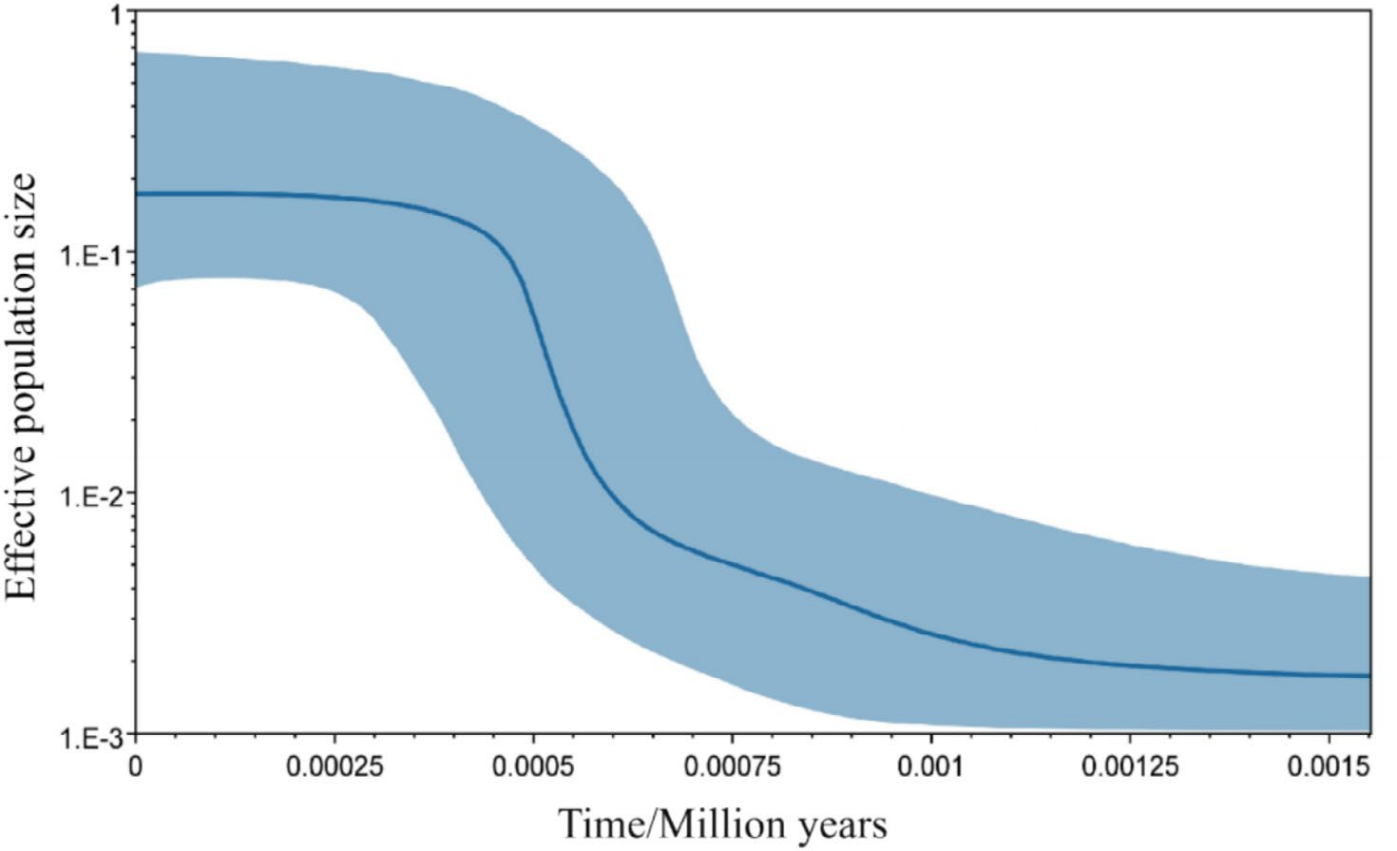
Bayesian skyline plot (BSP) of the *COI* + *Cytb* sequences of *Pastor roseus*.

Based on the mitochondrial *COI* and *Cytb* gene sequences, tests of molecular signatures of neutrality and base mismatch analysis were performed on 10 geographic populations of *P. roseus* . For *COI*, Tajima's *D* test values of each population were negative and deviated from the neutral expectation, reaching *p* > 0.05 for most populations. A similar trend was found in Fu's *F*_S_, with significance reaching *p* < 0.05; however, for most populations (Table 6). For *Cytb*, Tajima's *D* values were negative and deviated from neutrality for every population, with *p* < 0.05 for most populations, while Fu's *F*_S_ values were significant (*p* < 0.05) for most populations (Table 7). The results of base mismatch analysis differed from the observed values, and the curve showed an unimodal mismatch distribution (Figure 5), suggesting that populations may have undergone population expansion or positive selection. Results of BSP showed (Figure 6) that the blue solid line represents the median population size, while the blue shaded area denotes the 95% posterior density interval (HPD). The overall approximate expansion time (*T*) indicates that the *P. roseus* population expansion occurred approximately 0.0015 Ma.

**TABLE 6:** The neutrality tests of 10 *Pastor roseus* populations based on *COI* gene sequences.

Statistics	TC‐1	TC‐2	TC‐3	HM‐1	HM‐2	MNS	YN‐1	YN‐2	YN‐3	HM2024	Mean	SD
Tajima's *D*	−0.85	−1.35	−1.03	−0.95	−1.03	−0.01	−1.06	−1.46	−1.29	−1.01	−1.00	0.40
Tajima's *D p* value	0.19	0.07	0.16	0.18	0.20	0.54	0.17	0.07	0.10	0.16	0.18	0.13
FS	−4.02	−2.21	−5.32	−6.65	−1.99	−1.08	−4.64	−2.86	−8.94	−8.07	−4.58	2.66
FS *p* value	0.01	0.05	0.00	0.00	0.05	0.25	0.00	0.05	0.00	0.00	0.04	0.08

*Note:* TC_1 through YN_3 are all samples from 2025; HM2024 is a sample from 2024.

**TABLE 7:** The neutrality tests of 10 *Pastor roseus* populations based on *Cytb* gene sequences.

Statistics	TC‐1	TC‐2	TC‐3	HM‐1	HM‐2	MNS	YN‐1	YN‐2	YN‐3	HM2024	Mean	SD
Tajima's *D*	−1.80	−1.28	−1.76	−1.54	−1.43	−0.47	−1.15	−1.77	−1.62	−1.52	−1.44	0.40
Tajima's *D p* value	0.02	0.10	0.03	0.05	0.02	0.39	0.15	0.03	0.04	0.07	0.09	0.11
FS	−3.53	−3.34	−6.44	−7.44	−3.03	−0.93	−4.47	−2.71	−9.76	−6.26	−4.79	2.64
FS *p* value	0.01	0.01	0.00	0.00	0.01	0.25	0.01	0.03	0.00	0.00	0.03	0.08

*Note:* TC_1 through YN_3 are all samples from 2025; HM2024 is a sample from 2024.

## 4 Discussion

Genetic diversity is the basis for the survival and evolution of populations; the richer the genetic diversity, the greater the potential for survival, reproduction, and expansion of a population and the greater the ability of that population to resist and adapt to environmental changes (Vergeer and Postuma 2022). Haplotype diversity (*H*_d_) and nucleotide diversity (π) are important indicators for evaluating the genetic diversity of populations, and the larger their values are, the higher the genetic diversity of the population is (Li et al. 2018). Haplotype diversity of each population was categorized into a low category (0–0.5) and a high category (0.5–1.0) according to a threshold value of 0.5 (Li et al. 2022); populations were also classified into one of three categories of nucleotide diversity (Xiao et al. 2024), which considers the proportion of haplotypes in the population and is a more precise indicator of genetic diversity (Neigel and Avise 1993; Ma et al. 2019). The average number of nucleotide differences (*K*) indicates the number of different nucleotides at the same position in the same locus from two different individuals. When comparing two gene sequences, the nucleotide difference number reflects the similarities and differences between them. A higher nucleotide difference number indicates more differences between two sequences, while a lower number indicates greater similarity between them (Starikov 2023). In this study, based on the sequences of two mitochondrial genes (*COI* and *Cytb*) of *P. roseus* in 10 geographic groups, both genes showed consistent base composition: C > A > T > G, with A + T content exceeding C + G. This aligns with vertebrate mtDNA characteristics (37%–50% C + G) (Nei and Koehn 1983; Stanton et al. 1993) and closely matches related migratory birds (*Phylloscopus* spp., *Grus nigricollis* ) (Wang 2020; Wang et al. 2018; Johns and Avise 1998; Hochachka and Mommsen 2005; Zhuang et al. 2013; Xie et al. 2024; Zhang et al. 2018). The haplotype diversity values were 0.976 ± 0.006 and 0.944 ± 0.018, respectively. The nucleotide diversity values were 0.00316 ± 0.00016 and 0.00292 ± 0.00021, respectively. The mean nucleotide difference number values were 4.896 and 3.334, respectively. Both genes demonstrated high haplotype diversity and moderate nucleotide diversity in *P. roseus* . Xiao et al. (2024) found that eight genes in 11 geographic populations of chestnut‐backed short‐footed bulbuls ( *Hemixos castanonotus* ) had high haplotype diversity and moderately high nucleotide diversity, indicating that this population had experienced long‐term cumulative genetic differentiation, has a long evolutionary history, and is rich in genetic variation. Zhang (2007) analyzed the mitochondrial DNA gene (*D‐loop*) of Eurasian tree sparrow ( *Passer montanus* ) and found that the π and *H*_d_ values were 0.0021 ± 0.0004, and 0.755 ± 0.057, respectively; this species had high haplotypic diversity but low nucleotide diversity, indicating that its population may have experienced a historical expansion but with a limited level of genetic diversity. In comparison, both haplotype diversity and nucleotide diversity of *P. roseus* were higher than those of tree sparrows, suggesting that *P. roseus* have higher genetic diversity, which helps them to adapt to different environmental conditions and resist external pressures, thus increasing the possibility of long‐term population survival.

Haplotype number (i.e., haplotype richness) reflects the genetic diversity of a population and serves as an important measure for assessing genetic variation within and between populations, while also providing insights into species migration patterns, evolutionary history, and genetic structure (Aktaş 2024; Klyosov 2018). Analysis of nucleotide variation among different geographic populations of *P. roseus* confirmed genetic differentiation across the 10 sampled groups. A total of 62 haplotypes were detected in the *COI* gene (15 shared) and 69 in the *Cytb* gene (11 shared). However, the level of differentiation did not meet subspecies criteria, indicating that all populations belong to the same species, with close kinship, frequent gene flow, and no significant geographic isolation. Notably, within the Hami breeding population, several mitochondrial haplotypes were shared across samples collected in both 2024 and 2025. Given that mitochondrial haplotypes are stably inherited through the maternal line, this pattern suggests that individuals carrying the same haplotype likely belong to the same maternal lineage and exhibit high interannual breeding‐site fidelity to the Hami region. This information is important for understanding the kinship, population differentiation, and historical evolution among different geographic groups of *P. roseus* (Zou et al. 2020).

The genetic differentiation index (*F*_ST_) and gene flow (*N*_m_) are important indicators for evaluating the genetic structure of populations (Wang et al. 2017). The value of *F*_ST_ can be utilized to evaluate the degree of genetic differentiation among populations; *F*_ST_ ≤ 0.05 indicates that there is a weak genetic differentiation among populations; 0.05 < *F*_ST_ ≤ 0.15 indicates that there is a moderate level of genetic differentiation among populations; *F*_ST_ > 0.15 indicates that there is a high level of genetic differentiation among populations (Wright 1978; Gao et al. 2020). In this study, most of the genetic differentiation among different populations of rosy starling samples was not significant, which was consistent with the inferred haplotype network. The AMOVA results indicated that most of the genetic variation in *P. roseus* occurred within the populations. Genetic variation among populations was low in *P. roseus* , which explains why their genetic differentiation was not significant. Populations 2025TC‐1 and 2025YN‐3 had the greatest *F*_ST_ values (*COI*, 0.19085; *Cytb*, 0.12009) among populations, suggesting that genetic differentiation was greatest between these two populations and was significantly higher than that of other populations. It is hypothesized that this may be owing to the fact that Tacheng and Yining are separated by the Tian Shan Mountain range (Wang 2006) and further suggests that the degree of genetic differentiation among populations may be influenced by both distance and environmental isolation. In addition, the *N*_m_ values of all pairs of populations were all > 1.0, and their haplotype network relationship maps also showed a mixed haplotype distribution pattern, indicating that although the genetic exchange among populations was restricted, this restriction may have occurred recently. The isolation of the existing populations thus began too recently to lead to significant differentiation among groups. Multimodal mismatch distributions indicate that the shape of the gene tree is highly random, suggesting stable and balanced population dynamics, whereas unimodal mismatch distributions would indicate a high level of migration as populations have recently expanded or neighboring populations have expanded in their distribution areas (Zhan et al. 2023). In the present study, the distribution curves of nucleotide mismatch were relatively smooth, conforming to the expectation of unimodal curves associated with population expansion. Tajima's *D* values were not significant in both genes analyzed, while Fu's *F*_S_ values were significant, which indicated that there was a deviation from the neutral expectation of the *P. roseus* genes across the 10 geographic groups. These findings indicated that *P. roseus* Xinjiang experienced a large population expansion in the recent past, but with a more stable population size since then and no significant selective pressure. BSP results demonstrate a continuous decline in the effective population size of *P. roseus* beginning approximately 1500 years ago. Notably, between 1500 and 750 years ago, the population experienced a dramatic contraction, decreasing by nearly two orders of magnitude, after which it has persisted at a historically low level. The time frame of this recent population crisis aligns closely with the climatic deterioration associated with the transition from the Medieval Warm Period to the Little Ice Age across Eurasia, as well as a concurrent intensification of human activities. This concurrence suggests that both climatic and anthropogenic factors may have jointly contributed to the prolonged population bottleneck observed in *P. roseus*, as supported by Ahmed et al. (2013). The findings elucidate the underlying historical origins of the species' current vulnerability in genetic diversity, underscoring the importance of habitat protection.

Xinjiang is one of the regions in China with the most severe locust infestations (Gong 2001). There are many locust species and large numbers of locusts distributed in this province, which seriously endanger the grasslands of Xinjiang and affect the regional agricultural and animal husbandry economy (Yang et al. 2007). Each year, *P. roseus* migrates to Xinjiang in early May and returns to their wintering grounds in late August (Du et al. 2018), which is highly consistent with the occurrence period of grassland locusts in Xinjiang. The peak period of locust numbers occurs after the *P. roseus* chicks hatch. The parent birds can thus quickly obtain food to complete the brooding process and increase their success rate of reproduction (Wang et al. 2025). This phenomenon is the result of the combined effect of biological characteristics (high reproductive capacity, specialized diet) (Wang 2007), the special ecological conditions in Xinjiang (periodic locust outbreaks, warm and humid climate) (Yao et al. 2022), and human conservation management practices (artificial recruitment, engineering avoidance) (Tang et al. 2008). To some extent, these factors may explain why *P. roseus* populations expand during the breeding season. Genetic diversity improves the adaptability and survival capacity of species, which is essential for their long‐term survival and reproduction (Ran et al. 2023). *Pastor roseus* not only provides a low‐cost means of controlling locust damage, but also can overcome the problem of resistance of locusts to pesticides and still reduce their damage to crops (Shi and Tan 2019). Therefore, protecting the genetic diversity of *P. roseus* is of great importance for maintaining local biodiversity and ecological balance. In the actual conservation of *P. roseus* starlings, it is necessary to consider the genetic diversity and genetic differentiation among populations, which helps in formulating effective conservation measures. This study analyzed the genetic diversity and genetic structure of *P. roseus* from different geographical populations in Xinjiang at the molecular level. The research results provide empirical guidance and support for the rational utilization of rosy starlings to control grassland locusts.

## 5 Conclusion

Based on mitochondrial genetic markers, this study reveals that *P. roseus* population in Xinjiang exhibits high genetic diversity and active interpopulation gene flow, with overall weak genetic differentiation, indicating extensive genetic connectivity among subpopulations. However, some subpopulations have experienced a degree of differentiation due to geographical isolation. Historical population analyses suggest that the species has experienced expansion recently, but the effective population size has continued to decline over the past millennium, indicating that historical climatic and anthropogenic factors may have triggered population bottlenecks. As a key predator of locusts in Xinjiang's grasslands, the breeding period of *P. roseus* aligns closely with locust outbreak periods, and its population health is crucial for regional ecological balance and biological control. Therefore, protecting the genetic diversity of its habitats is essential for safeguarding the species' ecological functions and long‐term survival. Future research should integrate nuclear genetic markers and tracking technologies to further elucidate the dynamics of population structure and migratory ecology.

## Author Contributions

**Xixiu Sun:** data curation (equal), formal analysis (equal), investigation (equal), methodology (equal), software (equal), validation (equal), writing – original draft (lead). **Xiaojie Wang:** data curation (equal), formal analysis (equal), investigation (equal), methodology (equal), writing – original draft (lead). **Ran Li:** investigation (equal), validation (equal). **Huixia Liu:** software (equal). **Ye Xu:** software (equal). **Rong Ji:** project administration (equal). **Jun Lin:** investigation (equal). **Kun Yang:** investigation (equal). **Xiaofang Ye:** funding acquisition (lead), writing – review and editing (lead).

## Funding

This work was supported by Tianshan Talent Leading Talent Project of Xinjiang Uygur Autonomous Region, TSYCLJ0016. Tianshan Innovative Research Team of Xinjiang Uygur Autonomous Region, 2024D14006. Tianshan Young Talent Project for Outstanding Young Scholars of Xinjiang Uygur Autonomous Region, China, 2024TSYCCX0063.

## Ethics Statement

This study was conducted in accordance with the ethical guidelines of the Academic Morality and Ethics Committee of Xinjiang Normal University, and received approval with the review number [XJNU2024LLSC002]. All sampling and experimental procedures are strictly carried out in accordance with relevant guidelines and regulations.

## Conflicts of Interest

The authors declare no conflicts of interest.

## Supporting information


**Table S1:** TC_1 through YN_3 are all samples from 2025; HM2024 is a sample from 2024; The lower diagonal presents *F*_ST_ values, while the upper diagonal presents Nm values. An “Undetermined” annotation signifies an infinite Nm value.

## Data Availability

The original data is included in the Table S1.
